# The Non-Interventional PAZOREAL Study to Assess the Effectiveness and Safety of Pazopanib in a Real-Life Setting: Reflecting a Changing mRCC Treatment Landscape

**DOI:** 10.3390/cancers14225486

**Published:** 2022-11-08

**Authors:** Christian Doehn, Martin Bögemann, Viktor Grünwald, Manfred Welslau, Jens Bedke, Martin Schostak, Thomas Wolf, Rainer Ehneß, Elisa Degenkolbe, Stefanie Witecy, Peter J. Goebell

**Affiliations:** 1 Urologikum Lübeck, 23566 Lübeck, Germany; 2 Department of Urology, University of Münster Medical Center, 48149 Münster, Germany; 3 Department of Internal Medicine (Tumor Research) and Department of Urology, West German Cancer Center, University Hospital Essen, 45147 Essen, Germany; 4 Klinikum Aschaffenburg, Hämato-Onkologische Schwerpunktpraxis, 63739 Aschaffenburg, Germany; 5 Department of Urology, University Hospital Tübingen, 72076 Tübingen, Germany; 6 Department of Urology, Urooncology, Robot-Assisted and Focal Therapy, University Hospital Magdeburg, 39120 Magdeburg, Germany; 7 Outpatient Centre for Oncology, 01307 Dresden, Germany; 8 Novartis Pharma GmbH, 90429 Nürnberg, Germany; 9 APOGEPHA Arzneimittel GmbH, 01309 Dresden, Germany; 10 Department of Urology, University Hospital Erlangen, 91054 Erlangen, Germany

**Keywords:** pazopanib, renal cell carcinoma, real-world data, non-interventional study, time on drug, nivolumab, everolimus, trial-eligibility

## Abstract

**Simple Summary:**

Clinical trials have demonstrated the effectiveness of pazopanib as a primary treatment for metastatic renal cell carcinoma (mRCC). The approval of tyrosine kinase inhibitors and checkpoint-inhibitors represented further progress in the mRCC treatment landscape. Yet, with the recent changes in treatment options, there are scarce real-world data on these substances, including pazopanib. The PAZOREAL study investigated the effectiveness and safety of pazopanib (first-line), nivolumab (second-line), and everolimus (second- and third-line) in a real-life setting. This study included 376 mRCC patients who received first-line treatment with pazopanib and assessed the treatment’s effectiveness, safety, and resultant quality of life. The median time on the drug for the study population was 10.0 months; for primary treatment with pazopanib, it was 6.3 months. The median overall survival (mOS) for the entire study population was 35.9 months. No new safety signals were detected. PAZOREAL provides valuable real-world data for the primary treatment of mRCC with pazopanib.

**Abstract:**

The approval of tyrosine kinase inhibitors and checkpoint inhibitors represented a remarkable progression in the therapeutic landscape for the treatment of metastatic renal cell carcinoma (mRCC). Yet, in the ever-evolving landscape of mRCC treatment, real-world data on these agents, including pazopanib, are scarce. The non-interventional PAZOREAL study investigated the effectiveness and safety of pazopanib (first-line), nivolumab (second-line), and everolimus (second- and third-line) in a real-life setting. The multicentric study included 376 mRCC patients who received first-line treatment with pazopanib and assessed time on the drug (primary endpoint), overall survival, best responses, disease control rates, as well as safety signals and health-related quality of life. The median overall time on the drug was 10.0 months, with first-line pazopanib having a median time on drug of 6.3 months. The median overall survival was 35.9 months. The disease control rate for first-line pazopanib was 56.9%. No new safety signals were detected. PAZOREAL provides valuable real-world data for first-line treatment with pazopanib.

## 1. Introduction

Renal cell carcinoma (RCC) is considered the most common malignant tumor of the kidney and is diagnosed in about 17,300 patients per year in Germany [1]. At the time of diagnosis, most patients are between 60 and 80 years of age, with considerably more men being afflicted with RCC than women [2]. Clear cell RCC is by far the most common subtype, accounting for about 75% of all RCC cases [3]. When diagnosed, up to 16% of patients already have metastatic RCC (mRCC), and up to 30% recur after curative therapy [4,5].

The introduction of tyrosine kinase inhibitors (TKI), such as pazopanib, was considered a milestone in the first-line treatment of mRCC [6]. Treatment with everolimus, a serine threonine kinase inhibitor (mTOR), commonly followed as a second-line treatment [7,8]. In addition, the approval of nivolumab by the European Medicines Agency (EMA) in April 2016 paved the way for the routine clinical use of checkpoint inhibitors (CPI) for the treatment of mRCC [6,9]. Several CPI-based combination therapies have since been approved for the treatment of mRCC, and are currently considered the standard of care [10]. Despite these other treatment options, pazopanib is still recommended in guidelines as the first-line treatment for all types of RCC when the standard of care is not an option; it is still used in routine clinical practice [11,12].

To achieve a better understanding of the use of pazopanib in routine clinical practice, and the outcomes achieved in this setting, it is important to collect and evaluate real-world data. Additionally, efficacy and safety outcomes should be followed across subsequent treatments to gain information about the entire course of treatment. To date, real-life data for the treatment with pazopanib in first-line and sequential treatment are scarce. Although the safety and efficacy of pazopanib for the first-line treatment of mRCC had been evaluated in pivotal, randomized, double-blind, placebo-controlled, and multinational trials and other clinical studies [13,14,15], further real-world data are needed to evaluate the effectiveness, safety, tolerability, and quality of life (QoL) in routine clinical practice. Currently, we lack data that reflect the clinical use of pazopanib and associated outcomes within the constantly evolving treatment landscape of mRCC.

The non-interventional study called PAZOREAL, presented here, aims at providing some of the first insights into this knowledge gap.

## 2. Materials and Methods

### 2.1. Study Design and Patients

PAZOREAL is a prospective, multi-center, non-interventional observational study that evaluates the effectiveness, tolerability, safety, and QoL in mRCC patients that have been treated with pazopanib as first-line treatment and nivolumab or everolimus as second-line or third-line treatments. A total of 450 patients were planned to be enrolled from about 150 sites in Germany, including patients of oncologists in hospitals and outpatient clinics, as well as in independent oncology practices. PAZOREAL started in December 2015 (first patient enrolled) and lasted until February 2021 (end of observation). Eligible patients were adults with advanced or metastatic RCC and a life expectancy of at least 6 months who started first-line treatment with pazopanib or third-line everolimus no earlier than 8 weeks prior to giving informed consent. The current paper focuses on patients in routine care under first-line treatment with pazopanib at the start of the study. Data on patient demographics, vital signs, concomitant diseases, Karnofsky Performance Status (KPS) or ECOG score (Eastern Cooperative Oncology Group) [16], as well as disease history were assessed at baseline. If available, patient disease history comprised the primary diagnosis of RCC, including the type of histology, tumor staging, and risk assessment, according to the IMDC (International Metastatic RCC Database) Heng score [17,18]. Data concerning nephrectomy and metastasis were also documented at baseline.

### 2.2. Endpoints and Assessments

The primary endpoint of the study was the time on drug (ToD), defined as the time between the date of first and last administration of study medication in the respective treatment line. Further assessment of effectiveness was based on overall survival (OS). Clinical response rates were determined as SOC every 12 weeks during the course of the study and radiologically or clinically categorized by local investigators according to local clinical practice. Categories included progressive disease (PD), complete response (CR), stable disease (SD, i.e., non-CR, non-PD), and not evaluable.

In addition to the data collected at baseline, further assessments were performed at subsequent visits every 12 weeks throughout the study: ECOG score or KPS [16] and either administered dose of pazopanib (first-line) or nivolumab (second-line). If applicable, any dose modifications, treatment interruptions, or discontinuation of treatment were recorded together with corresponding reasons. Quality of life (QoL) was reported via the EQ-5D-5L questionnaire [19].

To assess for safety, all adverse events (AE), including serious AE (SAE), treatment emergent AE (TEAE), adverse drug reactions (ADR), and serious ADR (SADR), were documented for the study medications from the start of therapy until 30 days after completion of the treatment phase. Any AE that was temporally related to the study medication were considered TEAE. Toxicities were classified and documented according to common terminology criteria for adverse events (CTCAE).

Additional information about adverse events, disease progression, subsequent therapies, and survival status was recorded at follow-up visits every 6 months.

### 2.3. Statistical Analysis

Descriptive statistical methods were mainly used for the analysis and presentation of data. Time-to-event data, including OS and ToD, were estimated using the Kaplan–Meier method [20], and presented as median and quartiles together with the corresponding 95% confidence intervals (CI) and the frequency and percentage of events and censored cases. For ToD and OS, a sensitivity analysis with trial-eligible patients was performed [14,21,22]. Trial-eligibility was defined as not matching any of the three “trial-ineligibility criteria”, i.e., a definition based on Marschner et al. [22] and the exclusion criteria that were frequently applied in mRCC clinical trials during the TKI study period of the pivotal phase III trials [14,21]. These criteria included: a Karnofsky Performance Status < 70%, hemoglobin below the lower normal limit, and non-clear cell carcinoma. On the other hand, patients fulfilling at least one of these criteria were considered “trial-ineligible”.

The primary analysis population (i.e., full analysis set, FAS) of this paper included all patients for whom documentation started with first-line treatment with pazopanib (FAS-cohort I) and who received at least one dose of the observed drug. Patients for whom documentation started with third-line treatment with everolimus were not examined in this paper. Where applicable, results focused on the treatment course irrespective of the treatment sequence and in-detail results are presented for first-line treatment with pazopanib; detailed data of the specific treatment sequences are only presented in supplementary manner.

The secondary analysis population (i.e., safety analysis set, SAF) included all patients from FAS-cohort I who received at least one dose of the observed drugs and for whom at least one further post-baseline result (e.g., laboratory tests) was available. Any safety-related analyses, such as those concerning any type of adverse events (AE), were based on the SAF.

## 3. Results

### 3.1. Study Population

**Figure 1:**
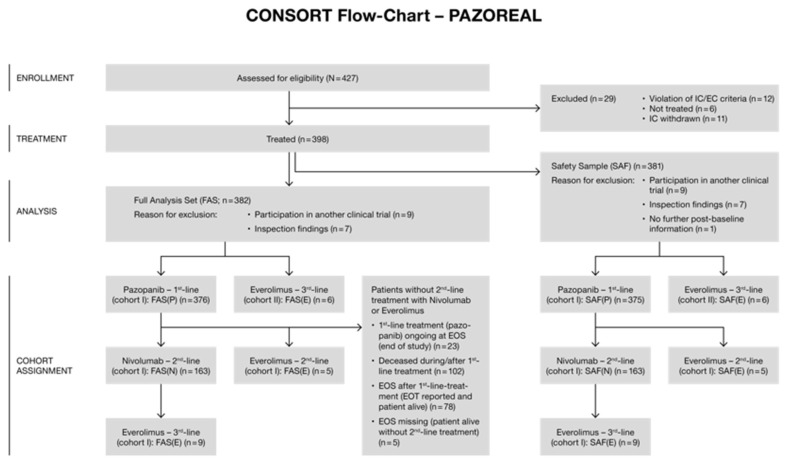
CONSORT flow chart for PAZOREAL with retrospective subset definition.

A total of 398 patients were treated in the study period; 16 of these patients were omitted from analysis (9 due to participation in another clinical trial; 7 due to inspection findings). In total, 382 study subjects were enrolled from 119 sites. Of these, 376 study subjects had documentation beginning with first-line treatment with pazopanib (i.e., FAS-cohort I; see Figure 1). The SAF of FAS-cohort I comprised 375 patients with first-line pazopanib treatment. Of these, 163 patients received nivolumab as second-line treatment, 5 patients received everolimus as second-line treatment, and 9 patients received everolimus as third-line treatment.

At baseline, patients in FAS-cohort I were aged between 38 and 89 years, with a median age of 69.7 years. Just over one third of all patients (*n* = 132, 35.1%) were aged <65 years at the beginning of the study, whereas 244 patients (64.9%) were 65 years or older. Two thirds of the patients were male (*n* = 257, 68.4%; see Table 1 for all baseline characteristics). The median observation time for FAS-cohort I was 44.6 months (95% CI: 43.2–47.1).

The most common initial doses of pazopanib were 800 mg (*n* = 248, 66.0%) and 400 mg (*n* = 100, 26.6%). Other initial doses between 200 and 600 mg were administered in considerably fewer patients (14 patients; less than 3.7% of the total study population). Doses were adjusted in 250 patients (66.5%), increased in 1 patient (0.4%), reduced in 227 patients (90.8%), and interrupted in 114 cases (45.6%).

For first-line treatment with pazopanib, the reasons for discontinuation of treatment were documented for 349 patients (92.8%). The most common reason for the end of treatment was progression of the disease (*n* = 197, 52.4%), followed by therapy-related toxicity (*n* = 51, 13.6%), non-therapy related AE (incl. SAE) (*n* = 22, 5.9%), death (*n* = 22, 5.9%), and other reasons such as patient’s wish or investigator’s decision. For 23 patients (6.1%), treatment with pazopanib was ongoing after the end of the observation period, and for 4 patients, there was no documented reason for discontinuation of treatment.

After first-line treatment with pazopanib, 163 patients (43.4%) received second-line treatment with nivolumab. The reasons for a discontinuation of second-line treatment with nivolumab were documented for 143 patients (87.7%); details can be found in Table S1. As the focus of this paper is on the results for pazopanib, the second-line nivolumab treatment is only briefly touched upon in the main text; it can be viewed in more detail in the supplementary section. Due to the small number of patients treated with everolimus in second- (5 patients) or third-line treatments (6 patients), results for sequential treatment with everolimus were omitted.

In FAS-cohort I (*n* = 376), 146 patients (38.8%) were deemed “trial-eligible” as they did not meet any of the three “trial-ineligibility criteria”. A total of 184 patients (48.9%) were classed as trial-ineligible. For 46 patients (12.2%), a response to at least one of the three “trial-ineligibility criteria” was missing, and therefore could not be assigned to one of the groups.

### 3.2. Effectiveness

#### 3.2.1. Time on Drug

The median overall time on drug (ToD) for FAS-cohort I was 10.0 months (95% CI: 8.5–11.7), from the start date of the first pazopanib administration until the end date of the last administration of any study medication (i.e., either first-line pazopanib, second-line nivolumab or everolimus, or third-line everolimus). The median ToD of pazopanib was 6.3 months (95% CI: 5.6–7.4), the median ToD of nivolumab was 4.8 months (95% CI: 3.7–6.5), and the median ToD of everolimus was 2.2 months (95% CI: 1.6–NA; second-line) and 3.6 months (95% CI: 0.6–11.2; third-line). The 6-month ToD-rate across all treatment lines was 66.7% (95% CI: 61.6%–71.2%) and the 6-month ToD-rate of pazopanib was 52.2% (95% CI: 47.0–57.1). A third of patients were still receiving pazopanib 12 months after starting first-line treatment with pazopanib (*n* = 111, 29.5%). Of the remaining patients, 99 patients (26.3%) were deceased, 55 patients (14.6%) ended the study for reasons other than death, and 44 patients (11.7%) received second-line treatment with nivolumab. All other patients were either in-between treatments, in follow-up, or under observation without treatment.

Based on the sensitivity analysis, the median overall ToD was 11.3 months (95% CI: 9.2–14.3) for trial-eligible patients in FAS-cohort I; the median ToD of pazopanib was 7.7 months (95% CI: 6.1–9.0). Further details on nivolumab are listed in Table S2.

#### 3.2.2. Overall Survival

**Figure 2:**
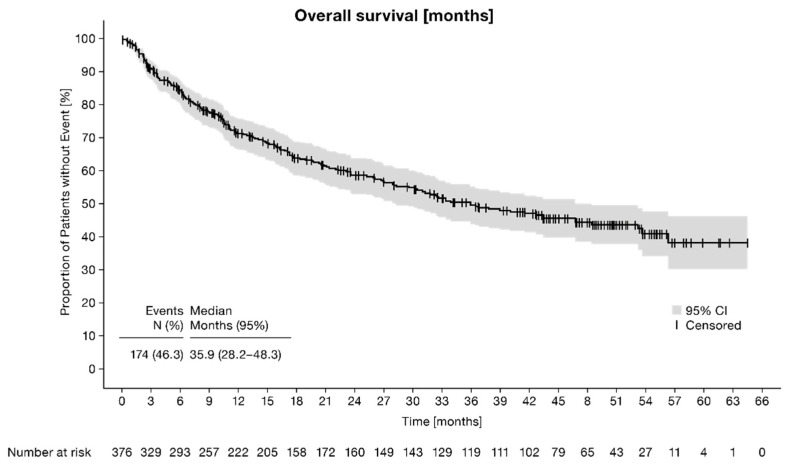
Overall survival of FAS-cohort I.

The median overall survival (OS) of FAS-cohort I was 35.9 months (95% CI: 28.2–48.3) (Figure 2). Of the 376 evaluable patients, 174 patients died (46.3%), and the remaining 202 patients (53.7%) were censored at the last date known alive. The sensitivity analysis based on trial-eligible patients revealed a median OS of 53.2 months (95% CI: 38.9-NA). Of the 146 evaluable patients, 59 patients died (40.4%), whereas the remaining patients were censored at the last date known alive (*n* = 87, 59.6%). However, the 12-month OS rates of FAS-cohort I and trial-eligible patients were comparable, at 71.5 (95% CI: 66.4–76.0%) and 77.9% (95% CI: 69.9–84.0%), respectively. Further sensitivity analyses of OS for FAS-cohort I were performed on sex and age (<65 years, ≥65 years) at start of therapy line (Table 2). The median OS of patients receiving first-line pazopanib and second-line nivolumab, compared with patients with other second-line therapies, is shown in Figure S1. 

#### 3.2.3. Best Response and Disease Control Rate

Based on radiological or clinical assessments by local investigators according to local clinical practices, 36 patients (9.6%, 95% CI: 7.0–13.0) receiving first-line treatment with pazopanib achieved a complete response (CR) as their best response, whereas stable disease (SD) was reported as the best response for 178 patients (47.3%, 95% CI: 42.4–52.4). Thus, the disease control rate (DCR), comprising patients with CR and SD, was 56.9% (95% CI: 51.9–61.8). PD was reported in 81 patients (21.5%, 95% CI: 17.7–26.0). The best response was assessed by radiologic assessment (259 patients, 68.9%) or clinical assessment (38 patients, 10.1%). No best response was evaluable for 2 patients, and in 79 patients (21.0%), no assessments were performed. The results of the response analysis for the 163 patients receiving nivolumab as second-line treatment are shown in Table S2.

### 3.3. Safety

Under first-line treatment with pazopanib, 1923 TEAE were documented in 337 patients (89.9%). Of those events, 1038 (54.0%) were judged to be related to pazopanib, which occurred in 270 patients (72.0%). The most common TEAE included diarrhea, nausea, and stomatitis (Table 3). Furthermore, there were 368 grade 3/4 TEAE (19.1%) occurring in 179 patients (47.7%), out of which 151 grade 3/4 TEAE (7.9%) in 95 patients (25.3%) were assessed as being related to pazopanib (Table 4). A total of 129 patients (34.4%) experienced TEAE that led to the discontinuation of treatment, and 66 of these patients (17.6%) were assessed to have TEAE related to pazopanib. In addition, 75 fatal TEAE (3.9%) were reported, of which 3 events (0.2%), occurring in 3 patients (0.8%), were judged as being related to pazopanib by the respective investigators. For each (0.3%) respective patient, the following reasons were reported: death without witnesses, disease progression, and neoplasm progression.

In 120 out of the 163 patients (73.6%) receiving second-line treatment with nivolumab following first-line treatment with pazopanib, a total of 400 TEAE were reported; further details on nivolumab are listed in Table S3. 

### 3.4. Quality of Life

**Figure 3:**
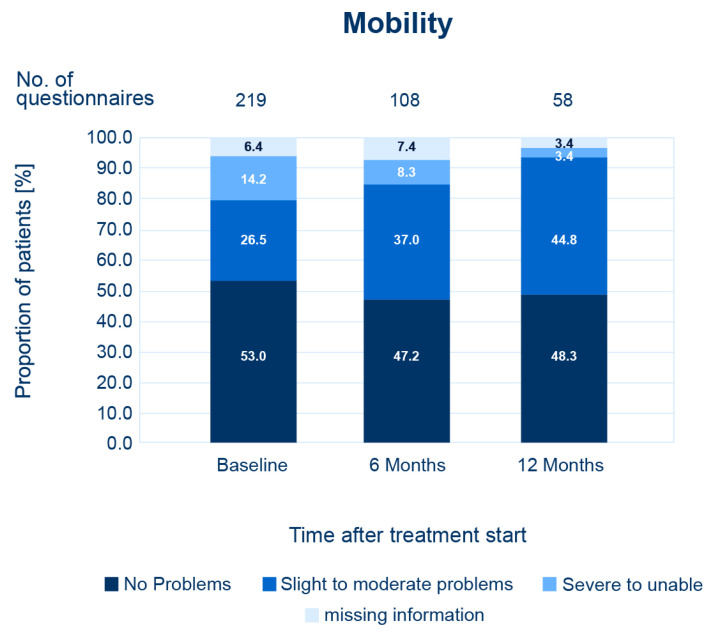
Quality of life under pazopanib for the “mobility” dimension at baseline and after 6 and 12 months of treatment. Percentages refer to the number of returned questionnaires. For other dimensions, see Figures S2–S5.

Among patients receiving first-line pazopanib treatment, 279 patients (74.2%) fulfilled the inclusion criteria for assessment of quality of life (QoL) with the EQ-5D-5L questionnaire—i.e., patients consented to data collection via questionnaire. Out of the 229 questionnaires handed out to patients, 219 (78.5%) questionnaires were available for analysis at baseline. Questionnaires from 58 patients (20.8%) were returned for analysis after 12 months of treatment. The EQ-5D-5L scores generally remained unchanged under treatment. After 12 months, most patients (*n* = 49, 84.5%) experienced no problems regarding self-care, whereas more patients reported problems ranging from “slight” to “severe problems/extreme discomfort” for the dimensions “Usual activity” (*n* = 34, 58.6%) and “Pain/Discomfort” (*n* = 37, 63.8%). About half of the patients reported no problems for the dimensions “Mobility” (*n* = 28, 48.3%) and “Anxiety/Depression” (*n* = 31, 53.4%) (Figure 3).

It should be noted that return rate of questionnaires was low. Therefore, results need to be interpreted with caution and should be understood as a description of the situation, rather than used for generalization. Likewise, for nivolumab as second-line treatment, there was also a low return rate of questionnaires. However, EQ-5D-5L scores generally remained unchanged. Further details on second-line nivolumab are listed in Table S4. 

## 4. Discussion

The results from the non-interventional PAZOREAL study presented here offer first insights into the routine care of mRCC with first-line treatment with pazopanib.

As the primary endpoint, the median ToD for patients receiving first-line treatment with pazopanib was more than 6 months, under routine clinical conditions. Specifically, the median ToD was 6.3 months for first-line pazopanib treatment in FAS-cohort I and median ToD for trial-eligible patients was 7.7 months. This is similar to published results from clinical studies with a ToD of 7.4 months in the pazopanib arm of VEG105192 [13,21], 8.1 months in the overall results for pazopanib of the COMPARZ-study [14,23], and a median ToD of 8.4 months for Asian patients and 7.2 months for non-Asian patients in a subsequent Asian vs. non-Asian subgroup analysis [24]. Overall, this indicates that real-world data are comparable to randomized controlled trial (RCT) settings.

Importantly, although non-interventional studies, including PAZOREAL, cannot derive any conclusions on the superiority of a treatment, they also have certain advantages compared with clinical trials. As data are coming from a less homogeneous patient pool, subpopulations of the patient group can give valuable insights into treatment options for special patient groups, e.g., older patients [25]. One prominent difference between the current real-world data population and those of published RCT studies was the age of the patients. In PAZOREAL, the median age of patients receiving first-line treatment with pazopanib was higher, with a median age of 69.7 years, compared with the median age of 59 (VEG105192) and 65 years (VEG107769) in other clinical trials [13,21,26]. Crucially, despite this difference, results of PAZOREAL are comparable to those of the clinical studies. Still, real-world studies have inherent limitations, such as low internal validity, various biases, and less quality control in data collection, and these factors must be considered when interpreting the results [25]. 

OS analyses showed a median OS of close to three years; as such, the median OS was longer compared with previous data from the COMPARZ study [23]. Additionally, more than half of the patients achieved either CR or SD. The CR rate under pazopanib was nearly 10%, much higher than expected from previous studies with CR rates of about 0.3 [21] or 0.4% [14]. This could be due to evaluation bias between study physicians as the assessment was not standardized. The CR must therefore be interpreted with caution and should be considered as a clinical complete response rate.

Furthermore, our results suggested that pazopanib was well-tolerated and no new safety signals were detected in PAZOREAL, making the safety profile comparable with findings of clinical studies [27,28]. Additionally, PAZOREAL provided valuable real-world data on trial-ineligible patients, as these patients were not represented in clinical studies in the initial pivotal phase III trials of sunitinib and pazopanib [14,21], despite making up a notable proportion of mRCC patients [29]. It is important to note that criteria on trial ineligibility have changed over time.

Whereas data on ToD is an important effectiveness endpoint of a therapy, it cannot be used to draw conclusions about the well-being of patients on the drug; accordingly, we also examined the quality of life of patients. However, considering the low return rates of the QoL questionnaires, these results must be regarded with caution, as they may be biased and may not be representative of the entire study population. Nevertheless, the results—as a non-generalizable description of the data—are in line with previous findings from pivotal studies that showed no discernible changes in QoL over the course of treatment with pazopanib [21]. Despite the low return rates of questionnaires, PAZOREAL added valuable information to the growing dataset on treatment strategies for mRCC, by assessing health-related QoL in mRCC patients in a real-world setting under treatment with pazopanib. This is particularly important given the lack of data on QoL.

The real-world assessment of routine practice also revealed that Heng scores and MSKCC risk scores were recorded for less than a quarter of all patients. Although the assessment of MSKCC risk scores was still used, further refinement has since been introduced with the IMDC score [11].

Although not the standard of care, pazopanib is approved as the first-line treatment for mRCC when drug combinations including CPI cannot be used and is still recommended in current guidelines [10,11,12]. The approval of nivolumab for the second-line treatment of mRCC provided a new treatment option for patients with disease progression after first-line treatment with pazopanib [30]. PAZOREAL shows that this sequence is already being used in clinical practice in Germany, as the majority of patients received nivolumab after pazopanib. This is also in line with clinical trials and previous analyses from PAZOREAL [31,32,33]. However, examination of differences between treatment sequences is still needed.

## 5. Conclusions

PAZOREAL provides real-world data from a study population encompassing patients receiving first-line pazopanib treatment in routine clinical care. Results from this non-interventional study support findings from clinical studies suggesting that pazopanib is an effective and safe first-line treatment for patients with mRCC who are not eligible for IO-based combinations. Crucially, this was true even though the real-world data showed a less homogenous patient pool than clinical studies; for instance, patients in PAZOREAL were older than in published clinical studies. With the approval of targeted substances for the treatment of mRCC, treatment options have increased and allowed for a more directed approach tailored to patient needs. PAZOREAL provides routine data on the use of pazopanib as the first-line treatment for mRCC.

## Figures and Tables

**Table 1:** Baseline characteristics of FAS-cohort I.

Characteristic	FAS-Cohort I, *n* = 376
Sex, *n* (%)	
Women	119 (31.6%)
Men	257 (68.4%)
Median age (range), in years	69.7 (38.5–89.2)
Age group at start of treatment with pazopanib	
<65 years	132 (35.1%)
≥65 years	244 (64.9%)
Median weight at baseline (range), in kg	79.0 (42.0–160.0)
Median BMI at baseline (range), in kg/m^2^	26.4 (16.8–58.4)
Number of “trial-eligible” patients, *n* (%) *	146 (38.8%)
Median time interval from primary diagnosis of RCC to first administration of pazopanib (range), in months	11.0 (0.2–339.3)
ECOG performance status, *n* (%)	
0	197 (52.4%)
1 (good)	104 (27.7%)
2 (moderate)	40 (10.6%)
3 (poor)	1 (0.3%)
4 (completely disabled)	1 (0.3%)
Not done/Missing	32 (8.5%)/1 (0.3%)
Histology, *n* (%)	
Clear cell	304 (80.9%)
Non-clear cell	38 (10.1%)
Unknown	34 (9.0%)
Patients with tumor in both kidneys at primary diagnosis, *n* (%)	16 (4.3%)
Metastatic or non-metastatic disease at enrollment, *n* (%)	
Metastatic disease	353 (93.9%)
Non-metastatic disease	23 (6.1%)
Number of metastatic sites, *n* (%)	
0	23 (6.1%)
1–3	322 (85.6%)
4–6	31 (8.2%)
5 most frequent localization of metastases, *n* (%)	
Lung	218 (58.0%)
Bone	96 (25.5%)
Liver	61 (16.2%)
Lymph nodes, regional	53 (14.1%)
Lymph nodes, distal	45 (12.0%)

* Defined as patients fulfilling none of the three ‘trial-ineligibility criteria’: (1) Karnofsky Performance Status <70%; (2) hemoglobin < lower limit of normal; (3) non-clear cell carcinoma histology.

**Table 2:** Sensitivity analyses for OS of FAS-cohort I on subgroup sex and age.

	Sex		Age at Start of Therapy Line	
Variable	Women	Men	<65 Years	≥65 Years
Patients (*n*)	119	257	132	244
Events *n* (%)	64 (53.8%)	110 (42.8%)	69 (52.3%)	105 (43.0%)
Median [95% CI]	31.2 [19.7–35.9]	46.7 [26.9–56.3]	30.4 [23.0–43.4]	43.4 [29.5-NA]
12-month OS rate [95% CI]	67.7% [58.1–75.5]	73.3% [67.1–78.5]	69.6% [60.7–76.9]	72.5% [66.1–77.9]

**Table 3:** Related TEAE of CTCAE severity grade 1/2 under first-line pazopanib with an occurrence of at least 5%.

Primary System Organ Class	Preferred Term	SAF, *n* = 375
**Patients with any event**		250 (66.7%)
Gastrointestinal disorders	Patients with any event	167 (44.5%)
	Diarrhea	116 (30.9%)
	Nausea	60 (16.0%)
	Stomatitis	19 (5.1%)
General disorders and administration site conditions	Patients with any event	75 (20.0%)
	Fatigue	47 (12.5%)
Skin and subcutaneous tissue disorders	Patients with any event	75 (20.0%)
	Hair color changes	33 (8.8%)
Nervous system disorders	Patients with any event	71 (18.9%)
	Dysgeusia	39 (10.4%)
Metabolism and nutrition disorders	Patients with any event	40 (10.7%)
	Decreased appetite	35 (9.3%)
Vascular disorders	Patients with any event	34 (9.1%)
	Hypertension	25 (6.7%)

Adverse event terms were encoded according to MedDRA version 20.0.

**Table 4:** Related TEAE of CTCAE severity grade 3/4 under first-line pazopanib occurring in at least 5 patients.

Primary System organ Class	Preferred Term	SAF, *n* = 375
**Patients with any event**		95 (25.3%)
Investigations	Patients with any event	28 (7.5%)
	Gamma-glutamyl transferase increased	8 (2.1%)
	Alanine aminotransferase increased	5 (1.3%)
	Aspartate aminotransferase increased	5 (1.3%)
Vascular disorders	Patients with any event	28 (7.5%)
	Hypertension	16 (4.3%)
	Hypertensive crisis	9 (2.4%)
Gastrointestinal disorders	Patients with any event	19 (5.1%)
	Nausea	7 (1.9%)
	Diarrhea	6 (1.6%)
General disorders and administration site conditions	Patients with any event	11 (2.9%)
	Fatigue	5 (1.3%)

Adverse event terms were encoded according to MedDRA version 20.0.

## Data Availability

Data available upon request to the corresponding author or sponsor of the clinical study.

## Supplementary Materials

The following supporting information can be downloaded at: https://www.mdpi.com/article/10.3390/cancers14225486/s1, Figure S1: Kaplan–Meier plot of overall survival under nivolumab, compared with other substances; Figure S2: Quality of life under pazopanib regarding the “self-care” dimension at baseline and after 6 and 12 months of treatment; Figure S3: Quality of life under pazopanib regarding the “usual activity” dimension at baseline and after 6 and 12 months of treatment; Figure S4: Quality of life under pazopanib regarding the “pain/discomfort” dimension at baseline and after 6 and 12 months of treatment; Figure S5: Quality of life under pazopanib regarding the “anxiety/depression” dimension at baseline and after 6 and 12 months of treatment; Table S1: Main reasons for discontinuation under second-line nivolumab; Table S2: Time on drug (ToD), best response, and disease control rate (DCR) under second-line nivolumab; Table S3: Summary of treatment-emergent adverse events (TEAEs) under second-line nivolumab; Table S4: Quality of life under second-line nivolumab at baseline and after 6 and 12 months of treatment.
