# Supplementary material for: The Non-Interventional PAZOREAL Study to Assess the Effectiveness and Safety of Pazopanib in a Real-Life Setting: Reflecting a Changing mRCC Treatment Landscape
Source: Cancers (Basel). 2022 Nov 8;14(22):5486. doi: 10.3390/cancers14225486 (PMC9688275; doi:10.3390/cancers14225486)
Supplement: Supplementary file 1 [file cancers-14-05486-s001.zip › cancers-2007842-supplementary.pdf]

*Supplementary Data*

# The Non-Interventional PAZOREAL Study to Assess the Effectiveness and Safety of Pazopanib in a Real-Life Setting: Reflecting a Changing mRCC Treatment Landscape

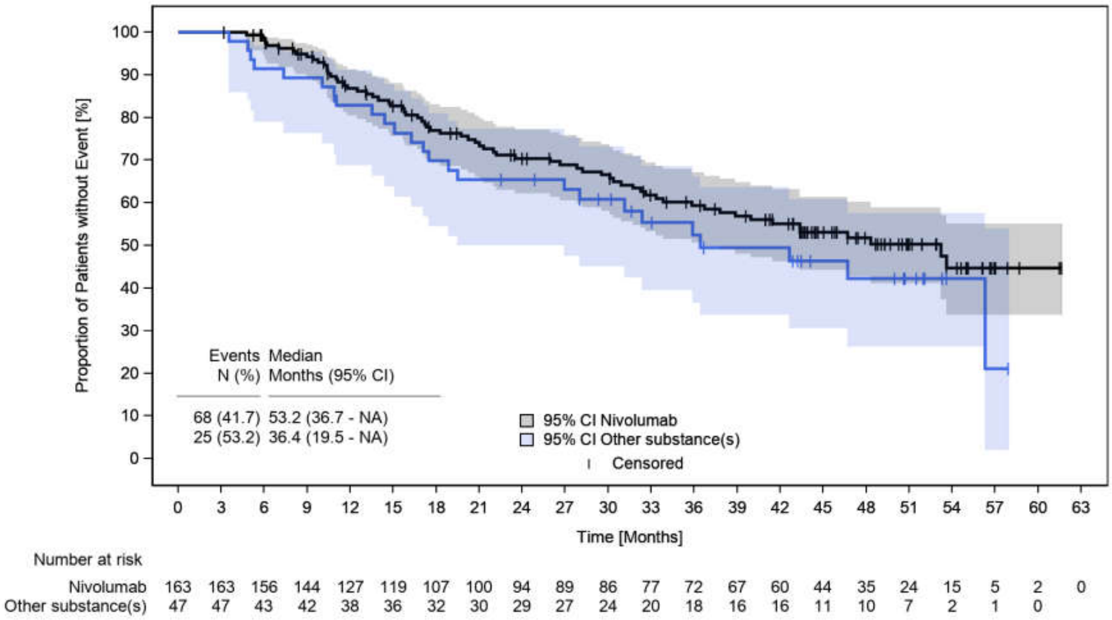


**Figure S1.** Overall survival of patients treated with nivolumab.

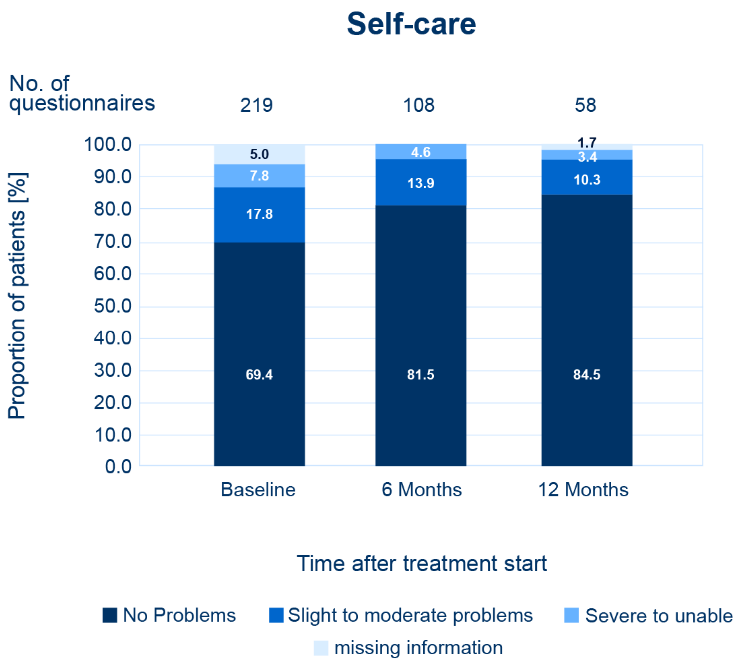


**Figure S2.** Quality of life under pazopanib regarding the dimension "self-care" at baseline and after 6 and 12 months. Percentages refer to the number of returned questionnaires.

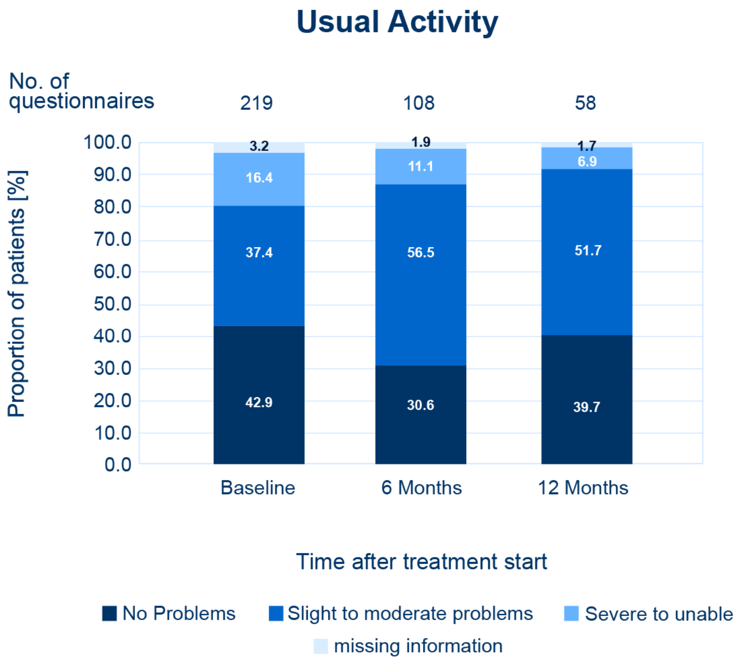


**Figure S3.** Quality of life under pazopanib regarding the dimension "usual activity" at baseline and after 6 and 12 months. Percentages refer to the number of returned questionnaires.

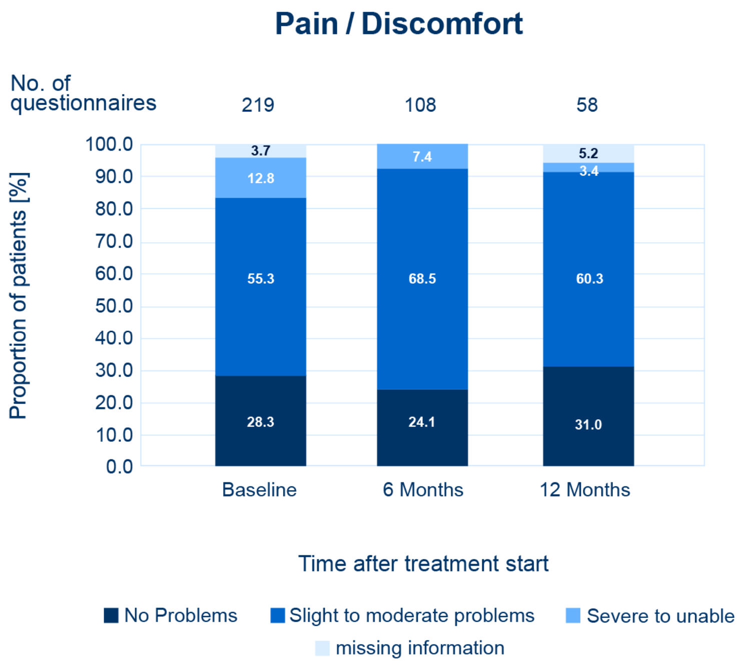


**Figure S4.** Quality of life under pazopanib regarding the dimension "pain / discomfort" at baseline and after 6 and 12 months. Percentages refer to the number of returned questionnaires.

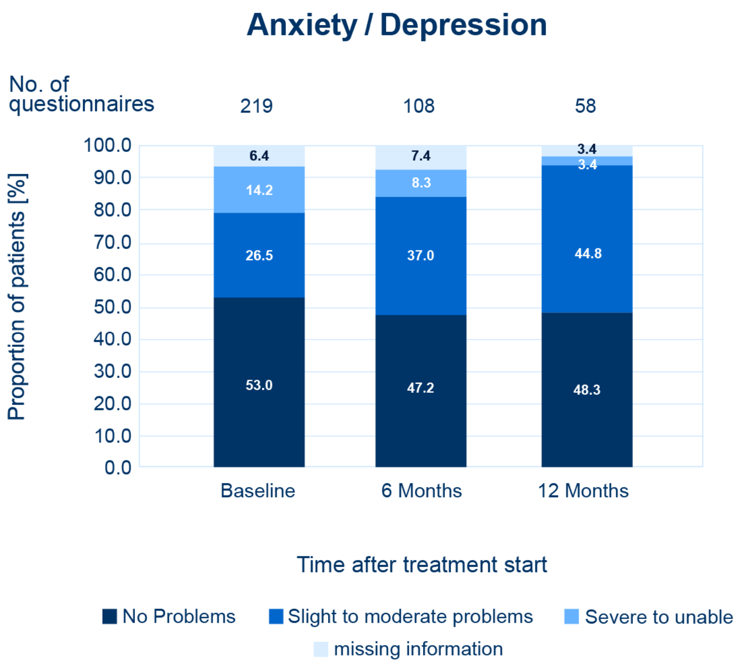


**Figure S5.** Quality of life under pazopanib regarding the dimension "anxiety / depression" at baseline and after 6 and 12 months. Percentages refer to the number of returned questionnaires.

**Table S1.** Main reasons for discontinuation under 2nd-line nivolumab.

| Reason for Discontinuation | Patients under 2nd-line Nivolumab, *n* = 143 |
|---|---|
| Progression of the disease, *n* (%) | 87 (53.4%) |
| Therapy-related toxicity, *n* (%) | 14 (8.6%) |
| Death, *n* (%) | 11 (6.7%) |
| Non-therapy related adverse events (AE) incl. serious AE and other reasons, *n* (%) | 7 (4.3%) |
| Investigator's decision (not toxicity, not therapy-related), *n* (%) | 7 (4.3%) |
| Patient's wish (not toxicity, not therapy-related), *n* (%) | 7 (4.3%) |
| Lost to follow-up, *n* (%) | 2 (1.2%) |
| Non-compliance, *n* (%) | 1 (0.6%) |
| Other reasons, *n* (%) | 7 (4.3%) |
| Missing, *n* (%) | 1 (0.7%) |

Reasons for discontinuation of the 2nd-line treatment with nivolumab were documented for 143 out of 163 patients (87.7%). For 19 patients (11.7%) treatment with nivolumab was ongoing after the end of the observation period.

**Table S2.** Time of drug (ToD), best response and disease control rate (DCR) under 2[nd]-line nivolumab.

| Endpoint | Patients under 2nd-line Nivolumab, *n*=163 |
|---|---|
| Median ToD, in months (95% CI) | 4.8 (3.7–6.5) |
| For trial-eligible patients | 3.9 (3.1–6.7) |
| 6-month ToD-rate, % (95% CI) | 43.9% (36.1%–51.1%) |
| For trial-eligible patients | 42.2% (30.0%-53.8%) |
| Best response, *n* (% [95% CI]) | |
| Complete response (CR) | 10 (6.1% [3.2–11.0]) |
| Stable disease (SD) | 61 (37.4%; [30.4–45.1]) |
| Progressive disease (PD) | 56 (34.4% [27.5–41.9]) |
| Assessment of best response, *n* (%) | |
| Radiologic assessment | 110 (67.5%) |
| Clinical assessment | 17 (10.4%) |
| No assessment done | 36 (22.9%) |
| DCR (patients with CR and SD), % (95% CI) | 43.6% (36.2–51.2) |

**Table S3.** Summary of treatment-emergent adverse events (TEAEs) under 2nd-line nivolumab.

| TEAE | Patients, *n*=163 | Cases, *n*=400 |
|---|---|---|
| TEAE, *n* (%) | 120 (73,6%) | 400 (100.0%) |
| TEAE grade 1/2, *n* (%) | 96 (58.9%) | |
| Related to nivolumab, *n* (%) | 40 (24.5%) | |
| Gastrointestinal disorders | 14 (8.6%) | |
| Diarrhea* | 9 (5.5%) | |
| Skin and subcutaneous tissue disorders | 13 (8.0%) | |
| TEAE grade 3/4, *n* (%) | 53 (32.5%) | 86 (21.5%) |
| Related to nivolumab | 23 (14.1%) | 28 (7.0%) |
| TEAE leading to discontinuation of treatment, *n* (%) | 38 (23.3%) | 56 (14.0%) |
| Related to nivolumab† | 15 (9.2%) | 19 (4.8%) |
| Fatal TEAE, *n* (%) | 22 (13.5%) | 22 (5.5%) |
| Related to nivolumab | 0 (0.0%) | 0 (0.0%) |

Adverse event terms were encoded according to MedDRA version 20.0.
*No other related TEAE grade 1/2 occurred in more than 5.0% of patients.
†No specific TEAE was reported for more than 2 patients (1.2%).

**Table S4.** Quality of life under 2nd-line nivolumab at baseline and after 6 and 12 months.

| EQ-5D-5L | Time After Treatment Start | | |
|---|---|---|---|
| | **Baseline** | **6 months** | **12 months** |
| **No. of returned Questionnaires, *n* (%)*** | 76 (52.1%) | 30 (20.6%) | 21 (14.4%) |
| Mobility, *n* (%) | | | |
| No problems | 36 (47.4%) | 15 (50.0%) | 7 (33.3%) |
| Slight to moderate problems | 22 (28.9%) | 8 (26.7%) | 10 (47.6%) |
| Severe to unable | 12 (15.8%) | 5 (16.7%) | 3 (14.3%) |
| Missing information | 6 (7.9%) | 2 (6.7%) | 1 (4.8%) |
| Self-Care, *n* (%) | | | |
| No problems | 48 (63.2%) | 20 (66.7%) | 13 (61.9%) |
| Slight to moderate problems | 22 (28.9%) | 7 (23.3%) | 5 (23.8%) |
| Severe to unable | 4 (5.3%) | 3 (10.0%) | 2 (9.5%) |
| Missing information | 2 (2.6%) | 0 (0.0%) | 1 (4.8%) |
| Usual Activity, *n* (%) | | | |
| No problems | 23 (30.3%) | 10 (33.3%) | 10 (47.6%) |
| Slight to moderate problems | 35 (46.1%) | 14 (46.7%) | 8 (38.1%) |
| Severe to unable | 16 (21.1%) | 6 (20.0%) | 3 (14.3%) |
| Missing information | 2 (2.6%) | 0 (0.0%) | 0 (0.0%) |
| Pain/Discomfort, *n* (%) | | | |
| No problems | 22 (28.9%) | 8 (26.7%) | 6 (28.6%) |
| Slight to moderate problems | 37 (48.7%) | 17 (56.7%) | 14 (66.7%) |
| Severe to unable | 15 (19.7%) | 5 (16.7%) | 1 (4.8%) |
| Missing information | 2 (2.6%) | 0 (0.0%) | 0 (0.0%) |
| Anxiety/Depression, *n* (%) | | | |
| No problems | 34 (44.7%) | 16 (53.3%) | 11 (52.4%) |
| Slight to moderate problems | 33 (43.4%) | 11 (36.7%) | 7 (33.3%) |
| Severe to unable | 6 (7.9%) | 3 (10.0%) | 2 (9.5%) |
| Missing information | 3 (3.9%) | 0 (0.0%) | 1 (4.8%) |

*146 patients (89.6%) qualified for the quality of life assessment. Percentages of the EQ-5D-5L dimensions refer to the number of returned questionnaires.
